# Infected omental cyst complicated with subacute intestinal obstruction and ileal erosion in a 2-year-old boy: a case report

**DOI:** 10.11604/pamj.2021.40.257.30755

**Published:** 2021-12-22

**Authors:** Hock Chin Chong, Hazlina Mohd Khalid, Nur Atiqah Mohd Hanifah, Vebster Jaffrey, Naveena Thiyagaraja, Firdaus Hayati

**Affiliations:** 1 Department of Surgery, Ampang Hospital, Ministry of Health Malaysia, Ampang, Selangor, Malaysia,; 2 Department of Paediatric Surgery, Sabah Women and Children Hospital, Ministry of Health Malaysia, Kota Kinabalu, Sabah, Malaysia,; 3 Department of Surgery, Faculty of Medicine and Health Sciences, Universiti Malaysia Sabah, Kota Kinabalu, Sabah, Malaysia

**Keywords:** Ileal erosion, intestinal obstruction, omental cyst, case report

## Abstract

An omental cyst is a rare intraabdominal pathology that can cause acute abdomen and intestinal obstruction among children. A 2-year-old boy presented with fever, bilious vomiting, abdomen distension and loose stool of acute onset. The chest and abdominal radiographs showed right pleural effusion and prominent small bowels with thickening walls respectively. Ultrasonography revealed a gastrointestinal duplication cyst. An exploratory laparotomy was done and found a 5x5 cm infected omental cyst, severely adhered to and eroded into the ileum causing a small perforation. Omental cyst excision and ileal primary repair were done. The final diagnosis was a benign omental cyst. Omental cyst is usually asymptomatic; however, symptomatic omental cyst should be cautious for bleeding, intestinal obstruction, infection, torsion and rupture.

## Introduction

An omental cyst is an uncommon intraabdominal pathology. It can mimic acute abdomen and intestinal obstruction among children in complicated cases [1,2]. The pathology behind omental cyst remains unclear but few theories have been proposed. Presentation of illness can be varied and non-specific in omental cyst but usually patients present with asymptomatic, chronic abdominal pain and acute abdomen [3,4]. Recommended initial imaging is ultrasound for children to help in diagnosis [5]. Excision of omental cyst is the standard operative management and the laparoscopy approach is preferred [3,5]. Here we discuss an infected omental cyst complicated with ileal erosion and subacute intestinal obstruction in a 2-year-old boy, the diagnostic dilemma and surgical management.

## Patient and observation

**Patient information:** a 2-year-old boy with no known medical illness, initially presented with fever for 8 days, after 2 days he developed bilious vomiting more than 10 episodes per day but still tolerate nourishing fluid, followed by abdomen distension and loose stool 5-10 episodes. Otherwise, none of his family members has any similar symptoms. He has no contact with tuberculosis patients.

**Clinical findings:** his vital signs are normal except fever spike daily. On inspection, he is active and not septic looking. His abdomen is distended but soft and not tender. There is no mass or hernia.

**Figure 1:**
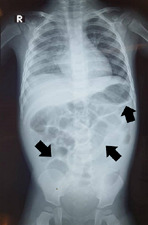
abdominal X-ray showed prominent small bowels with thickening bowel wall (arrow)

**Figure 2:**
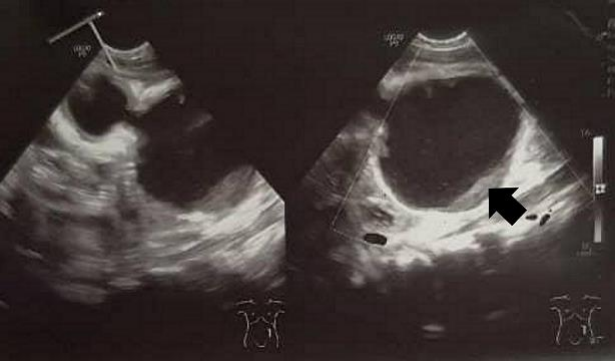
ultrasound imaging reported as duplication cyst with echogenic material within (arrow)

**Diagnostic assessment:** his haemoglobin is 7.6 g/dL, total white blood cell is 8.6 x10^3^/uL, the platelet is 380 x10^3^/uL, normal renal profile and liver function test. His chest radiograph noted right pleural effusion and the abdominal radiograph showed prominent small bowels with thickening walls without a transitional point (Figure 1). Ultrasound examination was performed and reported as a duplication cyst (Figure 2) with dilated small bowels and thicken wall but no free fluid.

**Figure 3:**
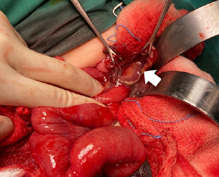
ruptured omental cyst (arrow) during mobilisation from dense adhesion with the surrounding structure

**Figure 4:**
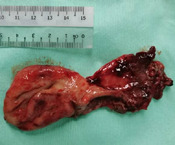
excised omental cyst

**Therapeutic intervention:** he was initially treated with intravenous ampicillin/sulbactam for enteritis and pneumonia. However, the condition was not improved despite medical treatment. Subsequently, an exploratory laparotomy was done and found a 5x5 cm infected omental cyst (arising from greater omentum) with 20 mL frank pus inside the thin cyst wall and adhered to surrounding small bowels. The cyst was severely adhered to and eroded into the ileum (Figure 3) and causes a small perforation of about 1 cm with a healthy edge. Excision of omental cyst (Figure 4) and ileal primary repair were done. Pus was sent for culture and grew *Escherichia coli* and *Klebsiella pneumoniae* which were sensitive to gentamicin, amikacin and cefepime. Histopathological examination reported as a benign omental cyst.

**Follow-up and outcomes:** the patient had recovered well after the surgery except for surgical site infection which needed intravenous gentamicin, wound saline flushing and dressing. After a week, the patient´s wound was healed and he discharged home.

**Patient perspective:** the patient was well taken care of during his hospitalization. The parents were relieved with treatment that was carried out on their child. They were happy to see such improvement from day one of admission until the day of discharge.

**Patient´s consent:** informed consent was obtained from the parents for us to utilize the pictures in this manuscript.

## Discussion

An intra-abdominal cyst can be grouped by location such as mesenteric, omental, solid organ and retroperitoneum [2]. Omental cyst is less common as compared to a mesenteric cyst. Omental and mesenteric cysts happen 1 in 20000 among children where only 2.2% of those cystic masses are omental cyst [1]. Only 1/4 of overall omental cyst report cases happen in kids below 10 years old [6]. Generally, omental cyst can be classified into congenital, infectious, traumatic and neoplastic cysts [7]. Since omental cyst is rare, the pathology remains unknown. Few pathogenesis theories had been proposed such as isolated benign ectopic lymphatic proliferation, lymphatic obstruction, failure of lymph channels to join venous system and degeneration of lymph node [6,7]. In this case, our patient is 2 years old and there is no risk factor such as trauma.

Diagnosis can be challenging where omental cyst could present in 3 ways: asymptomatic, chronic abdominal pain and acute abdomen [2]. When omental cyst becomes symptomatic, it is alarming and warns of complications such as cyst torsion, intracystic haemorrhage, infection, rupture, and intestinal obstruction [4]. Urgent surgical intervention is required in complicated omental cyst cases [4]. Initially, our patient was presented with loose stool, vomiting, fever and abdominal distension, hence treated as acute gastroenteritis. Those symptoms do not improve with antibiotics and bowel rest. Therefore, ultrasound was done later and become the key to the diagnosis. Some authors comment that ultrasound sometimes can misinterpret giant omental cyst as ascites [4]. Whenever in doubt, other option of imaging can be used is computed tomography (CT) scan and magnetic resonance imaging (MRI) [8,9]. Alternative imaging only should be used if necessary, as CT has a radiation problem while MRI needs sedation [6]. In our case, the scan has mistaken the cyst as an enteric duplication cyst as there is ileum erosion which appeared like having a connection between cyst and ileum. Duplication cyst is commonly found in the ileum which makes this preoperative diagnosis is crucial [10]. Nevertheless, intraoperatively noted that the omental cyst originated from greater omentum with thin wall cyst with severe adhesion and erosion into ileum causing small perforation and histopathology confirmed that it is an omental cyst. From the literature review, small bowel erosion with perforation never been reported as one of the omental cyst complications.

Complete excision is recommended for omental cyst and a laparoscopic approach is recommended [5]. Incomplete excision can lead to a recurrence of omental cyst [6]. The laparoscope strategy is to do a diagnostic examination, pull out the omental cyst through the umbilical port, drain the cystic fluid, and proceed with complete excision [5]. The conversion rate from laparoscope to open laparotomy can be as low as 6.4% [5]. We did not proceed with a laparoscopy in our patient because the abdomen is distended and bowels are dilated where the intraabdominal space is limited. Small perforation was found in the ileum about 1cm and covered by omental cyst. Omental cyst contained yellowish pus but there is no faecal material while the ileum perforation site contained greenish watery faeces, where it is unlikely to be a fistula. The ileal perforation edge is healthy and we decide to repair the perforation primarily. He is recovered well after the surgery. In case complete excision cannot be achieved, another option is partial excision followed by marsupialization or OK-432 injection [2]. This can bring down the recurrence rate from 0-13.6% [3]. Malignant transformation of the omental cyst is rare [1].

## Conclusion

Omental cyst is rare and usually asymptomatic. Symptomatic omental cyst should be aware and alert of its complications. In complicated omental cyst such as bleeding, intestinal obstruction, infection, torsion, and rupture, urgent surgical intervention is needed. We report here another omental cyst complication which is bowel erosion with perforation. Recommended diagnostic imaging is ultrasound. If in doubt with ultrasound finding, CT and MRI scan is an alternative. Complete excision of omental cyst is gold standard and laparoscope is preferred whenever feasible. If the complete excision is not possible, partial excision followed by marsupialization or OK-432 injection will be an option.
